# 

**Published:** 1923-12

**Authors:** 


### RECENT APPOINTMENTS.

### Medical Officers. Chaplains, &c.

Chalmers, Edith, M.B., Ch.B., Temporary Assistant M.O., Hull Corporation Asylum; Resident Assistant M.O., Hull Corporation Asylum. £300, with board.

Cellan-Jones, Katherine M., M.O., North Shields and Tynemouth Dispensary.

Haydock, W. S., M.D., D.P.H., Deputy Commissioner Medical Services, Belfast; Assistant M.O.H., County of Cumberland.

Hector, F. J., M.R.C.S., L.R.C.P., Second Assistant Resident M.O., Ham Green Hospital and Sanatorium.

Henriques, Stella; Assistant School M.O., Gillingham.

Massey, Arthur, M.B., Ch.B., Assistant M.O.H., Southampton Town and Port.

Phillips, Laurence, Second Assistant Resident M.O., Portsmouth (Trinity College, Dublin).

Pugh, Laura W., M.B., Ch.B., D.P.H., Assistant M.O., Monmouthshire County Council; M.O., Welsh Board of Health.

Sowden, George, M.R.C S., L R.C.P., D.P.H.; M.O.H. St. Pancras.

Sproat, H. B., M.D.; M.O.H., Rawdon5.

### Public Health and Hospital Appointments.

Birbeck, John S., Assistant Secretary, Nottingham General Hospital; Secretary, Salisbury Infirmary.

Braithwaite. Marian; Senior Health Visitor, Ashington.

Bath, Elsie (Queen Victoria Jubilee Nurses' Institute), Superintendent Surrey County Nursing Association.

Harrop, Florence Pemberton, School Nurse and Health Visitor, Doncaster; School Nurse and Health Visitor, Clitheroe.

Mark, Anne Maud Mary, Health Visitor, New Windsor.

Moore, Margaret, C.M.B., Emergency Health Visitor and School Nurse, Staffordshire County Nursing Association.

Pearce, H. K., Chief Clerk, Manchester Royal Infirmary; Secretary South Devon and East Cornwall Hospital, Plymouth.

Tarrant, A. J. M., Assistant Secretary, Royal Northern Hospital; Assistant to the General Superintendent and Secretary of Manchester Royal Infirmary.

Walton, James, Chief Sanitary Inspector, Manchester.

